# The Effect of the COVID-19 Pandemic on Myocardial Infarction Care in Kosovo

**DOI:** 10.3390/clinpract16090171

**Published:** 2026-09-18

**Authors:** Zarife Rexhaj, Besfort Kryeziu, Premtim Rashiti, Pranvera Ibrahimi, Arlind Batalli, Afrim Poniku, Margaritë Gjoka, Michael Henein, Shpend Elezi, Gani Bajraktari

**Affiliations:** 1 Clinic of Cardiology, University Clinical Centre of Kosova, 10000 Prishtina, Kosovo; drzariferexhaj89@gmail.com (Z.R.); pranvera_i86@hotmail.com (P.I.);; 2 Department of Internal Medicine, Medical Faculty, University of Prishtina, 10000 Prishtina, Kosovo; besfort.kryeziu@uni-pr.edu (B.K.); margarite.gjoka@gmail.com (M.G.); 3 Department of Public Health and Clinical Medicine, Umeå University, 901 87 Umeå, Sweden; 4 Imperial College, London W12 0BZ, UK; 5 Siena University, 53100 Siena, Italy; 6 University of Nicosia, Egkomi 2408, Cyprus

**Keywords:** acute myocardial infarction, COVID-19 pandemic, percutaneous coronary intervention, ST-segment elevation myocardial infarction, hospital admissions, in-hospital mortality

## Abstract

**Background and Aim:** Studies worldwide have documented a reduction in acute myocardial infarction (AMI) admissions during the COVID-19 pandemic. We aimed to assess AMI admissions, management, and in-hospital outcomes in Kosovo during the pandemic period. **Methods:** We conducted a retrospective single-centre study including all consecutive AMI admissions from 1 January 2018 to 31 December 2020 at the Clinic of Cardiology, University Clinical Centre of Kosovo, the only public tertiary primary percutaneous coronary intervention (PCI) centre in the country. The pre-pandemic (PP) period was defined as 1 January 2018–12 March 2020 and the ongoing pandemic (OP) period as 13 March–31 December 2020. Demographic, clinical, laboratory, angiographic, and outcome data were collected. In-hospital mortality was defined as death between admission and discharge. Interrupted time-series analyses were performed. **Results:** A total of 898 AMI patients were admitted during OP and 2955 during PP. Daily admissions decreased from 3.8/day to 3.05/day (*p* < 0.001). OP patients were younger, more often hypertensive and smokers, and more frequently male. Coronary angiography (81% vs. 76%, *p* = 0.001) and PCI (66% vs. 60%, *p* = 0.001) were more frequent during OP, while in-hospital mortality remained unchanged. Time-series analysis showed a sharp fall in admissions at OP (β = −12.66, *p* < 0.001) with gradual recovery, alongside an increase in PCI use (β = 0.184, *p* < 0.001) and stable mortality. The proportion of ST-segment elevation (STEMI) cases increased significantly during the pandemic period. Compared with the pandemic period, admission during the pre-pandemic period was associated with lower odds of undergoing PCI (aOR 0.81, 95% CI 0.69–0.95), while age ≥ 65 reduced the likelihood of PCI. **Conclusions:** AMI hospitalizations in Kosovo declined significantly during the COVID-19 pandemic. The pandemic cohort was younger, with the profile of admitted patients shifting toward younger age, male sex, and STEMI presentation. Despite fewer admissions, PCI use increased, while no statistically significant difference in in-hospital mortality was observed between the two periods. These findings describe changes in treatment patterns and short-term in-hospital outcomes during the pandemic but should not be interpreted as evidence of equivalent overall quality of AMI care.

## 1. Introduction

Since December 2019, the emergence of the coronavirus disease 2019 (COVID-19) has evolved into a global pandemic with an important impact on health care systems worldwide [1]. COVID-19 increased overall mortality mainly due to respiratory infection with its related complications [2]. In Kosovo, the first two COVID-19 cases were confirmed on 13 March 2020, when nationwide isolation and lockdown were implemented, as in other severely affected countries [3]. Beyond respiratory disease, the COVID-19 pandemic has impacted the diagnosis and treatment of acute myocardial infarction (MI). Several observational studies have demonstrated substantial drops in AMI admissions in different countries, often ranging from 20% to over 40% reductions [4,5,6,7,8,9,10]. The main reasons include patients’ hesitancy to seek care due to infection fears, lower referral rates, or even misdiagnosis [4,5,6]. These studies also reported delayed presentation times and increased in-hospital complications or mortality [10,11,12]. Additionally, rates of coronary revascularization were reported to decline in many regions during COVID-19 [5,13,14,15]. We aimed to study acute MI admissions and in-hospital outcomes during the COVID-19 lockdown period in Kosovo, and to analyze temporal trends and treatment patterns around the pandemic onset.

## 2. Methods

This single-centre cohort study included all consecutive patients with acute MI admitted from 13 March 2020 [the first COVID-19 case, ongoing pandemic (OP)] until 31 December 2020, at the Clinic of Cardiology of the University Clinical Centre of Kosovo in Prishtina, as the only public tertiary health care centre in Kosovo and the only public hospital in Kosovo which operates 24 h, 7 days a week for primary PCI. The control period was considered to be the period from 1 January 2018 to 12 March 2020 [pre-pandemic (PP)]. After receiving approval from the Ethical Council of the Kosovo Doctors Chamber (approval code 17/2021; approval date: 23 March 2021), data were collected by a researcher who administered a structured questionnaire for all patients with acute MI. Epidemiological data from patients’ records, including age, gender, risk factors (smoking, arterial hypertension, diabetes, dyslipidemia and family history of CAD), clinical data (cardiogenic shock, duration of hospitalization and outcome), biochemical data (glycemia at admission, cholesterol, triglycerides, urea, creatinine, hemoglobin and troponin), electrocardiogram, medications and data from an echocardiographic examination at admission were collected. In addition, conventional coronary angiographic data and the results of the interventional treatment were registered. Acute MI cases were identified according to the International Classification of Diseases, 10th Revision (ICD-10), using codes I21.0–I21.9, and the diagnosis was confirmed based on electrocardiographic findings and elevated cardiac troponin levels according to the laboratory reference range used at the time of admission. Patients were classified as STEMI or NSTEMI according to the presence of ST-segment elevation on the admission electrocardiogram. No additional exclusion criteria were applied beyond failure to meet the diagnostic criteria for acute MI. For patients with more than one AMI admission during the study period, only the first qualifying admission was included. In-hospital mortality was defined as death between the patient’s admission and discharge. Inclusion criteria were all patients with acute MI admitted to our clinical centre, including those who tested positive for COVID-19 by RT-PCR.

### Statistical Analysis

We presented categorical variables as *n* (%), whilst continuous variables were presented as mean ± SD. Missing data were handled using available-case analysis, hence no imputation was performed. We compared the categorical variables using Pearson’s χ^2^ test, and continuous variables using the independent-samples Student’s *t*-test. We performed an interrupted time-series (ITS) analysis on weekly aggregated data to evaluate changes associated with the onset of the COVID-19 pandemic. We assessed four outcomes, such as the weekly number of AMI admissions, the weekly proportion of AMI patients undergoing PCI, the weekly proportion of in-hospital deaths, and the weekly proportion of STEMI cases among all AMI admissions. We defined the intervention as the onset of the COVID-19 pandemic on 13 March 2020; because this date occurred during ISO week 11, ISO week 12 was considered the first complete post-intervention week. The segmented-regression model included continuous time, coded sequentially by study week, an intervention indicator coded 0 before and 1 after the interruption, whilst a post-intervention time variable coded 0 before the interruption and 1, 2, 3, etc. Thus, the model estimated the pre-pandemic temporal trend, the immediate change in level following the interruption, and the change in trend during the pandemic period. For inference, we used Newey–West standard errors with up to four weekly lags because evidence of serial correlation was identified in some models, using heteroskedasticity- and autocorrelation-consistent standard errors. All regression coefficients were reported with 95% CI and two-sided *p*-values. Due to proportional outcomes, regression coefficients represent absolute changes in proportion and are expressed as percentage-point changes (β × 100) in Table 1 to facilitate interpretation.

Logistic regression models were used to adjust for the factors that were linked to the chance of undergoing PCI, where the model covariates were age, sex, and AMI type. Weekly AMI hospitalization rates were smoothed using a 3-week moving average and were visualized to compare temporal patterns between the pre-pandemic and pandemic periods.

## 3. Results

This study included 898 AMI patients admitted in the OP period, and 2955 patients in the PP period. The number of hospitalized patients decreased from 3.68 admissions/day (2955 admissions over 802 days) during the PP period to 3.05 admissions/day (898 admissions over 294 days) during the OP period (*p* < 0.001). Patients in the OP period were younger (62.6 ± 11.4 vs. 64 ± 11.8 years, *p* = 0.002), with more smokers (53% vs. 51%, *p* = 0.036), fewer females (23% vs. 29%, *p* ˂ 0.001), and more hypertensives (84% vs. 67%, *p* ˂ 0.001) compared to the PP period. During the OP period, the proportion of STEMI admitted cases was significantly higher than in the PP period (64% vs. 52%, *p* < 0.001), and patients were more likely to undergo coronary angiography (81% vs. 76%, *p* = 0.001) and PCI (66% vs. 60%, *p* = 0.001). Their serum urea was lower (8.5 ± 5.3 vs. 9.26 ± 6 mmol/L, *p* = 0.004) compared to those admitted in the PP period. In-hospital mortality did not differ between the two periods, OP and PP (9.7% vs. 9.5%, respectively, *p* = 0.911) (Table 2).

### 3.1. Temporal Trends in AMI Admissions, Treatment and Outcomes

**Figure 1:**
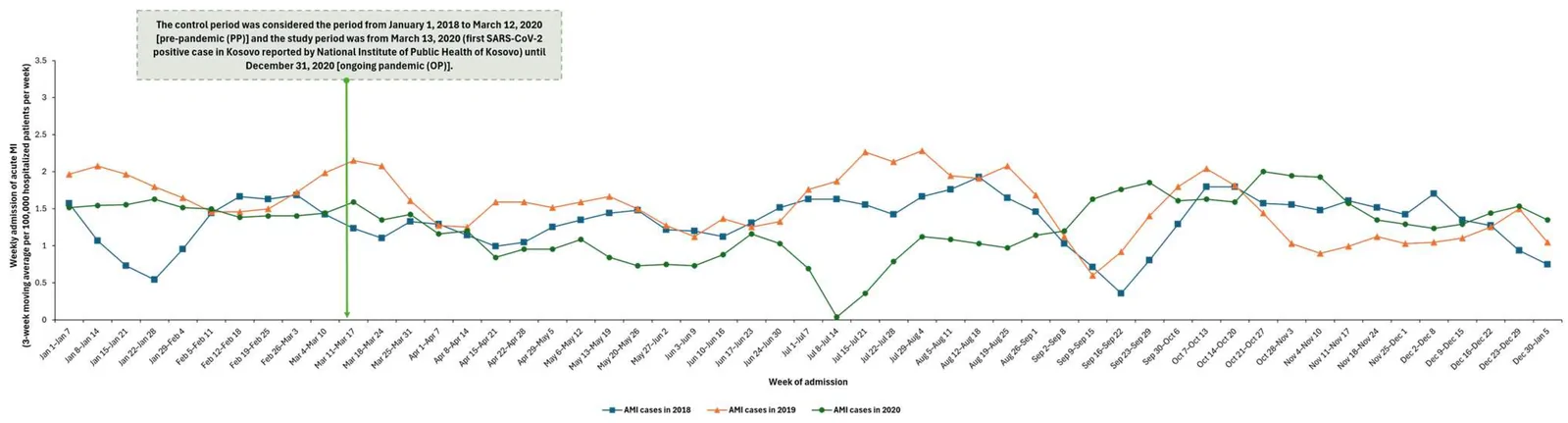
Three-week moving average of AMI admissions at the Clinic of Cardiology, University Clinical Centre of Kosovo, before and during the COVID-19 pandemic (1 January 2018 to 31 December 2020).

**Figure 2:**
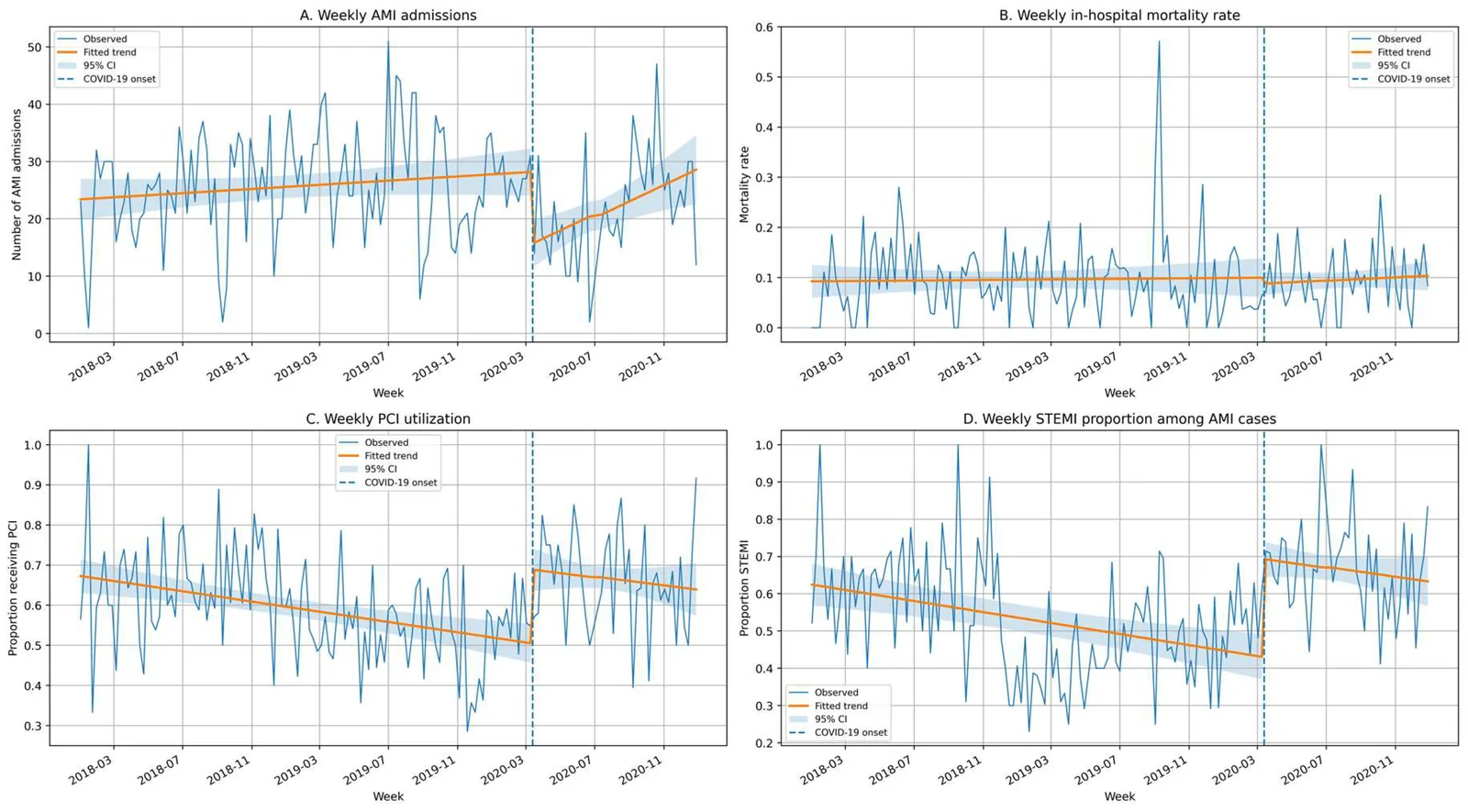
Interrupted time-series analysis of weekly AMI admissions, PCI utilization, in-hospital mortality, and STEMI proportion before and during the COVID-19 pandemic. The vertical line indicates the onset of the pandemic period (13 March 2020), whilst shaded areas indicate 95% CI around the fitted estimates. (**A**) Weekly AMI admissions; (**B**) Weekly in-hospital mortality rate; (**C**) Weekly PCI utilization; (**D**) Weekly STEMI proportion among AMI cases.

AMI admissions fell sharply following the onset of the pandemic (β = −12.66 admissions/week, 95% CI −18.65 to −6.67; *p* < 0.001), followed by a positive change in the weekly trend (β = 0.285, 95% CI 0.053 to 0.516; *p* = 0.016), indicating gradual recovery in admissions. The proportion of STEMI cases increased immediately (β = 0.263, 95% CI 0.185 to 0.342; *p* < 0.001), as did the proportion of patients undergoing PCI (β = 0.184, 95% CI 0.107 to 0.261; *p* < 0.001) (Figure 1 and Figure 2). No significant immediate change in the weekly proportion of in-hospital deaths was observed (β = −0.012, 95% CI −0.059 to 0.035; *p* = 0.615) Complete segmented-regression estimates are presented below. (Table 1).

### 3.2. Determinants of PCI Among AMI Patients

Table 3 shows adjusted odds ratios for factors associated with receiving PCI. With the pandemic period as the reference category, patients admitted during the pre-pandemic period had lower adjusted odds of receiving PCI (aOR 0.81, 95% CI 0.69–0.95; *p* = 0.009). Patients with STEMI were more likely to undergo PCI than those with NSTEMI (aOR 1.36, 95% CI 1.20–1.55, *p* < 0.001). Age ≥ 65 was associated with lower odds of PCI (aOR 0.73, 95% CI 0.64–0.84, *p* < 0.001). Male sex was not significantly associated (aOR 0.99, 95% CI 0.85–1.15, *p* = 0.879). These results indicate that, after adjustment for age, sex, and AMI type, PCI usage was lower during the pre-pandemic period than during the pandemic period.

## 4. Discussion

***Findings.*** Our study demonstrates a significant reduction in AMI hospitalizations in Kosovo during the COVID-19 pandemic compared with the pre-pandemic period, with daily admissions falling from 3.8 to 3.05 cases per day (*p* < 0.001). This decline was accompanied by a change in the clinical profile of admitted patients, who were more frequently male, young, hypertensive, and smokers. A larger proportion of admitted patients presented with STEMI, and coronary angiography and PCI were more frequently performed during the pandemic period. Despite that, in-hospital mortality remained unchanged. Time-series analysis confirmed an abrupt fall in admissions at the onset of the pandemic followed by gradual recovery, while PCI use increased. Multivariable analysis further showed that admission during the pandemic independently predicted receipt of PCI.

***Data interpretation and comparison with previous studies.*** The reduction in AMI admissions observed in our cohort is consistent with international reports from Europe and the United States, where declines of approximately 20–40% have been documented following the onset of COVID-19 restrictions [4,5,6,13,16]. Several mechanisms have been proposed, including fear of catching the infection, reduced mobility, decreased referrals, and potential misinterpretation of symptoms during lockdowns. Our data suggest that reduced access or delayed presentation rather than a true reduction in AMI incidence is the most likely explanation. We observed a lower proportion of women among patients admitted during the pandemic period; however, this finding reflects a difference in cohort composition rather than a demonstrated subgroup-specific decline in admissions. Similar sex-specific trends have been previously reported [5], raising concerns about barriers to acute cardiac care among female patients. Given the historically poorer outcomes of women with AMI, this finding highlights an important equity issue that warrants further investigation. Patients admitted during the pandemic were slightly younger than those in the pre-pandemic era, a pattern that has been described in some but not all registries [5,11,17,18]. This may partly reflect greater reluctance among older individuals to seek hospital care because of their higher perceived vulnerability to COVID-19. The higher prevalence of hypertension and smoking during the pandemic period likely reflects the risk profile of individuals who continued to access medical care despite lockdown conditions. The proportion of STEMI cases increased significantly during the pandemic, consistent with reports suggesting a shift toward more severe presentations [16,17]. Potential explanations include delayed care-seeking behaviour or reduced presentation of lower-risk NSTEMI cases. Notably, despite system pressures, the use of coronary angiography and primary PCI increased during the pandemic period in our centre. This contrasts with reports from some regions describing reduced revascularization activity [5,13], and may reflect the organization and resilience of AMI pathways in Kosovo. Our time-series analysis provides additional confirmation that the pandemic caused an abrupt decline in AMI admissions, with gradual recovery thereafter, while PCI use rose in parallel. Multivariable logistic regression showed that patients admitted during the pre-pandemic period had lower adjusted odds of receiving PCI compared with those admitted during the pandemic period. Taken together, our findings indicate that among patients who reached the hospital during the pandemic, PCI was performed more frequently than during the pre-pandemic period. No statistically significant difference in in-hospital mortality was observed between the two periods. This reflects a preserved capacity to deliver evidence-based reperfusion therapy despite pandemic-related constraints, demonstrating the robustness of national AMI care provision. The absence of a significant increase in in-hospital mortality is evident; however, it should be interpreted carefully when demonstrating equivalent outcomes or preserved overall quality of care.

***Study limitations.*** This study has several limitations that should be acknowledged. First, it was conducted at a single tertiary centre, although this is the only public PCI centre in Kosovo and therefore captures the majority of national AMI admissions requiring invasive evaluation. Nevertheless, patients treated in private institutions or who never presented to medical care could not be included, potentially underestimating the true burden of AMI during the pandemic period. Second, the retrospective design may introduce selection and information bias, although standardized data collection and complete hospital records minimize this risk. Third, we were unable to assess pre-hospital delays, total ischemic time, or causes of death after discharge, which may have provided further insight into the mechanisms underlying changes in presentation and outcomes. Fourth, differences in testing strategies, referral behaviour, and public health restrictions over time could not be fully accounted for. Finally, although interrupted time-series and multivariable analyses strengthen the findings, residual confounding cannot be excluded. Therefore, the results should be interpreted as observational associations rather than causal effects.

***Clinical implications.*** Our findings have several important clinical implications. The marked reduction in AMI admissions during the COVID-19 period suggests that a substantial proportion of patients may have delayed or avoided seeking medical care. This highlights the need for clear public messaging during public health emergencies to reinforce that symptoms of suspected AMI require urgent evaluation irrespective of pandemic conditions. The increased proportion of STEMI among presenting patients further supports the hypothesis of delayed presentation or selective attendance by higher-risk individuals. Despite pandemic-related strain on health services, PCI was performed more frequently among admitted AMI patients, while no statistically significant difference in in-hospital mortality was observed. These findings suggest that invasive treatment remained available to patients who reached the hospital; however, they should not be interpreted as demonstrating preserved or equivalent overall quality of AMI care. This underlines the importance of maintaining protected, uninterrupted acute cardiac care pathways during times of health care disruption. Future planning should ensure capacity and patient access to timely reperfusion therapy, while also addressing potential disparities in presentation and treatment, particularly among women and older patients.

## 5. Conclusions

During the COVID-19 pandemic in Kosovo, overall hospital admissions for acute myocardial infarction declined. The pandemic cohort was younger, included a lower proportion of women and NSTEMI patients, and had a higher proportion of STEMI cases. Despite fewer admissions, PCI was performed more frequently, while no statistically significant difference in in-hospital mortality was observed between the pandemic and pre-pandemic periods. These findings characterize changes in AMI presentation and treatment during the pandemic but do not establish equivalence in the overall quality of care.

## Figures and Tables

**Table 1:** Interrupted time-series estimates of changes in AMI admissions, treatment, and outcomes following the onset of the COVID-19 pandemic.

Outcome	ITS Term	β	95% CI	*p*-Value
AMI admissions	Pre-intervention trend	0.042	−0.016 to 0.100	0.156
	Immediate level change	−12.661	−18.619 to −6.702	<0.001
	Change in post-intervention trend	0.285	0.055 to 0.515	0.016
PCI proportion	Pre-intervention trend	−0.147 pp/week	−0.216 to −0.077	<0.001
	Immediate level change	+18.389 pp	+10.725 to +26.052	<0.001
	Change in post-intervention trend	+0.022 pp/week	−0.254 to +0.297	0.877
In-hospital mortality proportion	Pre-intervention trend	+0.006 pp/week	−0.050 to +0.063	0.823
	Immediate level change	−1.196 pp	−5.832 to +3.441	0.611
	Change in post-intervention trend	+0.033 pp/week	−0.089 to +0.155	0.589
STEMI proportion	Pre-intervention trend	−0.170 pp/week	−0.252 to −0.088	<0.001
	Immediate level change	+26.316 pp	+18.508 to +34.124	<0.001
	Change in post-intervention trend	+0.018 pp/week	−0.232 to +0.269	0.886

Footnote: For AMI admissions, β represents the absolute change in the number of weekly admissions. For proportional outcomes (PCI, in-hospital mortality, and STEMI), coefficients are expressed as absolute percentage-point (pp) changes (β × 100). The pre-intervention trend represents the weekly change before the pandemic, whilst the immediate level change represents the abrupt change at the intervention and finally the change in post-intervention trend represents the difference in slope after versus before the intervention.

**Table 2:** Baseline clinical characteristics of AMI patients.

Variable	Pre-Pandemic Period (*N* = 2955)	Ongoing Pandemic (*N* = 898)	*p* Value
Age (years, mean (SD))	64 ± 11.8	63 ±11.4	0.002
Female [*n*, (%)]	853 (29)	203 (23)	<0.001
Smoking [*n*, (%)]	1509 (51)	476 (53)	0.036
Diabetic [*n*, (%)]	1134 (39)	332 (37)	0.238
Arterial hypertension [*n*, (%)]	1976 (67)	753 (84)	<0.001
Glucose (mmol/L, mean ± SD)	9.65 ± 5.3	9.63 ± 5.3	0.919
Cholesterol (mmol/L, mean ± SD)	4.9 ± 1.4	5.1 ± 1.3	0.012
Triglycerides (mmol/L ± SD)	1.97 ± 1.5	1.5 ± 1.0	0.144
Urea (mmol/L, mean ± SD)	9.26 ± 6	8.5 ± 5.3	0.004
Creatinine (μmol/L, mean (SD))	118.4 ± 75	118 ± 63	0.901
RBC (×10^6^/mm^3^ mean (SD))	4.72 ± 0.8	4.73 ± 0.7	0.835
WBC (×10^3^/mm^3^, mean (SD))	10.5 ± 4.9	11.3 ± 3.2	<0.001
HCT % (mean (SD))	41.5 ± 7.2	41.3 ± 6.2	0.395
Hgb g/dL (mean (SD))	13.8 ± 2.3	13.6 ± 1.9	0.042
LV-EF% (mean (SD))	50 ± 8.6	51 ± 7.7	0.096
STEMI [*n*, (%)]	1527 (52)	574 (64)	<0.001
NSTEMI [*n*, (%)]	1428 (48)	324 (36)	<0.001
Patient undergoing coronary angiography [*n*, (%)]	2246 (76)	727 (81)	<0.001
PCI [*n*, (%)]	1773 (60)	593 (66)	<0.001
Mortality [*n*, (%)]	280 (9.5)	87 (9.7)	0.911

Abbreviations: AMI: acute myocardial infarction; STEMI: ST-segment elevation myocardial infarction; NSTEMI: non-ST-segment elevation myocardial infarction; PCI: percutaneous coronary intervention; LV-EF: left ventricle ejection fraction; RBC: red blood cell; WBC: white blood cell; HCT: hematocrit; Hgb: hemoglobin.

**Table 3:** Multivariable logistic regression of factors associated with PCI utilization among AMI patients.

Predictor	aOR	95% CI	*p*-Value
Pandemic period (reference)	1.00	-	-
Pre-pandemic period	0.81	0.69–0.95	0.009
Age ≥ 65 years	0.73	0.64–0.84	<0.001
Male sex	0.99	0.85–1.15	0.879
STEMI (vs. NSTEMI)	1.36	1.20–1.55	<0.001

## Data Availability

The datasets presented in this article are not readily available due to privacy and ethical restrictions. Requests to access the datasets should be directed to corresponding author.
